# Basic and clinical immunology – 3021. Inhibitory action of levocetirzine hydrochloride on eosinophil actication *in vitro*

**DOI:** 10.1186/1939-4551-6-S1-P197

**Published:** 2013-04-23

**Authors:** Atsuko Furuta, Kazuhito Asano, Harumi Suzaki

**Affiliations:** 1 Department of Otorhinolaryngology, School of Medicine, Showa University, Tokyo, Japan; 2 Physiology, Showa University, Japan

## Background

Histamine H_1_ receptor antagonists are used for the treatment of allergic disorders such as allergic rhinitis and atopic allergy with remarkable success. However, the influence of antihistamines on the function of eosinophils, which are the most important final effector cells in allergic diseases, is not well understood.

## Purpose

The influence of histamine H_1_ receptor antagonists on eosinophil functions was examined through the choice of levocetirizine hydrochloride (LH) *in vitro* and *in vivo*.

## Methods

BALB/c male mice (5 weeks of age) were intraperitoneally infected with 500 *Mesocestoides cortii* larvae. These mice were then treated with LH at a single dose of 0.1 mg/kg once a day, which was started on the day of infection. The percent of peripheral blood eosinophils and IgE levels were examined 21 days after infection. In the second experiments, eosinophils obtained from mice infected with *M cortii* were sensitized with *M.cortii*-specific IgE, and these sensitized eosinophils were stimulated with 10 ng/ml of *M. cortii* excretory antigen in the presence of LH for 24 h. MIP-1β, LTC_4_and RANTES levels in culture supernatants were examined by ELISA.

## Results

Oral administration of LH could not suppress both peripheral blood eosinophila and IgE hyper-production, which were observed in mice infected with *M cortii*. The addition of LH into cell cultures could suppress the ability of eosinophils to produce MIP-1β, LTC_4_ and RANTES, which were increased by SCF stimulation. The minimum concentrations of LH, which caused significant suppression of factor production, were 1.0μM for MIP-1β and LTC_4_, and 0.5 μM for RANTES.

## Conclusions

These results may suggest that LH exerts inhibitory effects on eosinophil activation and results in favorable modification of clinical status of pollinosis patients.

